# Plant Lectin Can Target Receptors Containing Sialic Acid, Exemplified by Podoplanin, to Inhibit Transformed Cell Growth and Migration

**DOI:** 10.1371/journal.pone.0041845

**Published:** 2012-07-23

**Authors:** Jhon Alberto Ochoa-Alvarez, Harini Krishnan, Yongquan Shen, Nimish K. Acharya, Min Han, Dean E. McNulty, Hitoki Hasegawa, Toshinori Hyodo, Takeshi Senga, Jian-Guo Geng, Mary Kosciuk, Seung S. Shin, James S. Goydos, Dmitry Temiakov, Robert G. Nagele, Gary S. Goldberg

**Affiliations:** 1 Graduate School of Biomedical Sciences, University of Medicine and Dentistry of New Jersey, Stratford, New Jersey, United States of America; 2 Department of Molecular Biology, School of Osteopathic Medicine, University of Medicine and Dentistry of New Jersey, Stratford, New Jersey, United States of America; 3 Proteomics and Biological Mass Spectrometry, Molecular Discovery Research, GlaxoSmithKline, Collegeville, Pennsylvania, United States of America; 4 Division of Cancer Biology, School of Medicine, Nagoya University, Nagoya, Japan; 5 Biologic and Materials Sciences, University of Michigan School of Dentistry, Ann Arbor, Michigan, United States of America; 6 New Jersey Institute for Successful Aging, University of Medicine and Dentistry of New Jersey, Stratford, New Jersey, United States of America; 7 Department of Surgery, Cancer Institute of New Jersey, New Brunswick, New Jersey, United States of America; 8 Department of Cell Biology, University of Medicine and Dentistry of New Jersey, Stratford, New Jersey, United States of America; Karolinska Institute, Sweden

## Abstract

Cancer is a leading cause of death of men and women worldwide. Tumor cell motility contributes to metastatic invasion that causes the vast majority of cancer deaths. Extracellular receptors modified by α2,3-sialic acids that promote this motility can serve as ideal chemotherapeutic targets. For example, the extracellular domain of the mucin receptor podoplanin (PDPN) is highly *O*-glycosylated with α2,3-sialic acid linked to galactose. PDPN is activated by endogenous ligands to induce tumor cell motility and metastasis. Dietary lectins that target proteins containing α2,3-sialic acid inhibit tumor cell growth. However, anti-cancer lectins that have been examined thus far target receptors that have not been identified. We report here that a lectin from the seeds of *Maackia amurensis* (MASL) with affinity for *O*-linked carbohydrate chains containing sialic acid targets PDPN to inhibit transformed cell growth and motility at nanomolar concentrations. Interestingly, the biological activity of this lectin survives gastrointestinal proteolysis and enters the cardiovascular system to inhibit melanoma cell growth, migration, and tumorigenesis. These studies demonstrate how lectins may be used to help develop dietary agents that target specific receptors to combat malignant cell growth.

## Introduction

Over 1 in 4 people are diagnosed with cancer at some point in their life [1]. These cancers are the leading cause of death of men and women under 85 years old [2]. Tumor cell motility contributes to metastatic invasion that causes the vast majority of cancer deaths [3], [4]. Extracellular receptors that promote this motility can serve as ideal chemotherapeutic targets [5]–[9]. Expression levels of receptors with α2,3-sialic acid residues are closely associated with the invasive and metastatic potential of many cancers [10]–[12] including skin cancer [13], [14]. Podoplanin (PDPN) represents one of these receptors.

PDPN is a unique transmembrane receptor that promotes tumor cell motility. PDPN expression can be induced by tumor promoters including TPA, oncogenic Ras, and Src [15]–[17]. For example, we have reported that Src utilizes the focal adhesion adaptor protein Cas to induce PDPN expression in order to promote tumor cell migration [15]. PDPN regulates the activities of effectors including ezrin, Rho, and Cdc42 to mediate filopodia formation and promote tumor cell migration, invasion, and metastasis [5], [18]–[22].

PDPN is found at the invasive front of many tumors, which is consistent with its role in promoting malignant invasion [5], [20], [23]. For example, PDPN expression is strongly induced in about 40% of breast cancers [20], [24], 50% of oral cancers [23], [25], [26], and 80% of skin cancers [27], [28]. The bulk of the PDPN protein, about 150 amino acids, lies outside of the cell and could serve as an ideal target to combat cancer invasion and metastasis [5], [29].

The extracellular domain of PDPN is highly *O*-glycosylated with sialic acid, α2,3 linked to galactose [5], [30]. PDPN is activated by endogenous ligands that bind to these extracellular carbohydrate moieties [31]–[33] to induce tumor cell motility and metastasis [20], [33]–[35]. Thus, blocking this interaction should inhibit malignant progression. For instance, compounds blocking the action of galectins, which activate mucin receptors, can inhibit tumor cell metastasis [36], [37].

Antibodies against PDPN can inhibit lung metastasis of transformed cells that express PDPN [38], [39]. However, antibody therapy presents challenges in administration and toxicity [40], [41]. Since PDPN is modified by α2,3-sialic acid, it can be targeted by specific lectins [10], [12]–[14], [42].

Unlike antibodies, lectins can survive digestive processing and retain biological activity [43]–[46]. Indeed, dietary lectins can block the action of endogenous pro-metastatic lectins (such as galectins or selectins) to inhibit tumor cell growth [37], [45]–[47]. For example, although its targets and mechanisms are not yet defined, Mistletoe lectin (viscumin) binds proteins containing α2,3-sialic acid, has undergone successful clinical trials, and is widely used to treat melanoma in Europe [48]–[50]. However, anti-cancer lectins that have been examined thus far target receptors that have not been identified.

Here, we show that a lectin with affinity for *O*-linked carbohydrate chains containing sialic acid binds to PDPN to inhibit transformed cell growth and motility at submicromolar concentrations. This approach demonstrates how lectins may be used as dietary agents that target specific receptors to combat malignant cell growth.

## Results

### MASL targets PDPN on Src transformed cells

**Figure 1:**
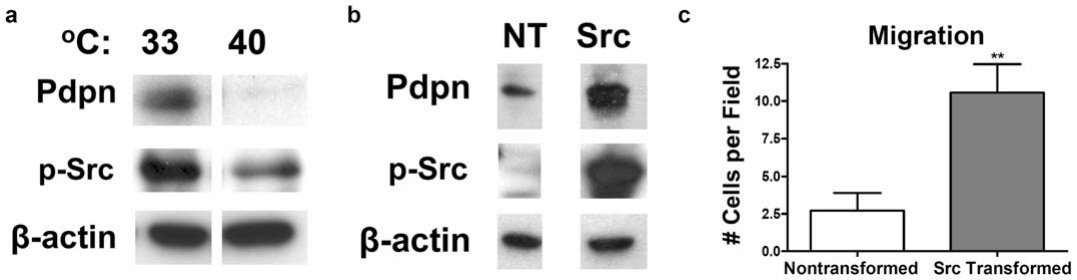
Src activation induces Pdpn expression and cell migration. (**a**) PDPN, active Src kinase (phosphorylated at Y-416), and β-actin were detected by Western blotting of protein (20 µg per lane) from LA-25 cells (NRK cells containing temperature sensitive v-Src) grown overnight at 33°C (permissive temperature) or 40°C (non-permissive temperature). (**b**) PDPN, active Src (phosphorylated at Y416), and β-actin were detected by Western blotting of protein (15 µg per lane) from nontransformed cells or Src transformed mouse embryonic cells (MEFs) as indicated. (**C**) Wound healing migration assays were performed on confluent monolayers of Src transformed or nontransformed cells. Data are shown as the number of cells that migrated into a 300×300 micron area along the center of the wound in 24 hours (mean + SEM, n = 7). Double asterisks indicate p<0.01 compared to untreated Src controls.

We have recently reported that the Src tyrosine kinase utilizes the adaptor protein Cas/BCAR1 to augment PDPN expression in order to promote tumor cell motility [15]. PDPN is also profoundly involved in the ability of transformed cells to escape contact normalization (growth control by surrounding nontransformed cells) and “break out” of their microenvironment to become malignant or metastatic [15]. Consistent with these previous reports, Src transformed cells expressed more PDPN (see **Figure 1a and 1b**) and migrated more than nontransformed controls (**Figure 1c**). As described above, the extracellular domain of PDPN is *O*-glycosylated with sialic acid, α2,3 linked to galactose. Scholl et al have reported that PDPN associates with lectin from seeds of the legume tree *Maackia amurensis* (MASL) [30], which has an affinity for *O*-linked carbohydrate chains containing sialic acid [51], [52]. These observations led us to investigate the ability of MASL to target PDPN on transformed cells.

**Figure 2:**
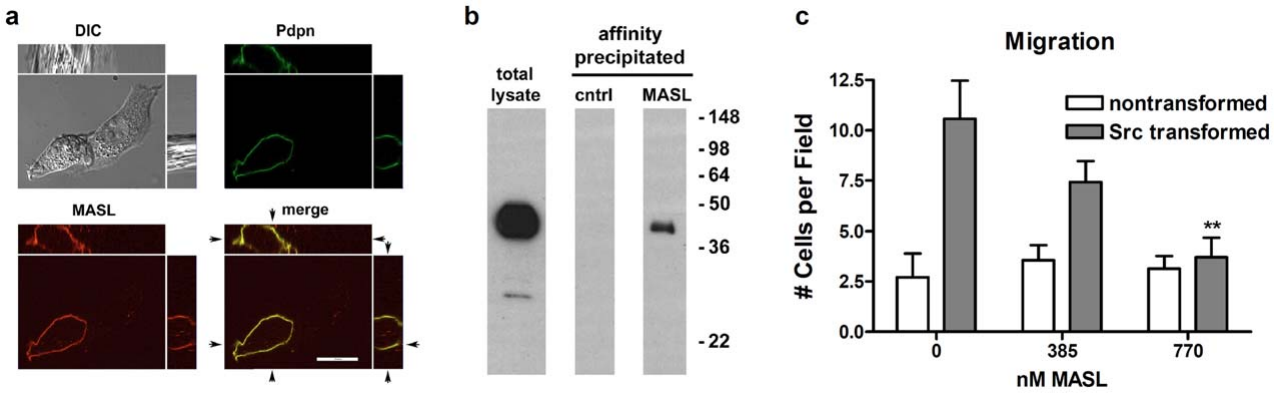
MASL associates with PDPN and inhibits migration of Src transformed cells. (**a**) Src transformed cells were exposed to MASL conjugated to HiLyte Fluor TR (red), and PDPN was detected by immunofluorescence microscopy (green). Colocalization of lectin and PDPN (yellow) is apparent in merged images, including orthogonal views of the z-axis which is 14 microns thick (bar = 20 microns). (**b**) Protein from Src transformed cells (750 μg) was precipitated with agarose beads linked to MASL, or control beads, and examined for PDPN by Western blotting. Cell lysate (15 μg/lane) were also examined as indicated. (**c**) Wound healing migration assays were performed on confluent monolayers of cells treated with concentrations of MASL as indicated. Data are shown as the number of cells that migrated into a 300x300 micron area along the center of the wound in 24 hours (mean + SEM, n = 7). Double asterisks indicate p<0.01 compared to nontransformed cells.

As shown in **Figure 2a**, MASL associated with PDPN on the membrane of Src transformed cells. Note that MASL did not target the membrane of cells that did not express PDPN, which, along with functional studies described below, attests to its preferential targeting. In addition, MASL associated with PDPN in lysates from transformed cells during affinity precipitation experiments as shown in **Figure 2b**.

### MASL inhibits Src transformed cell growth and migration

We hypothesized that binding of MASL to PDPN would inhibit transformed cell migration. As shown in **Figure 2c**, MASL significantly inhibited transformed cell migration, with 385 nM and 770 nM inhibiting cell migration by over 25% and 50% respectively. In contrast, MASL did not inhibit the migration of nontransformed cells in a dose dependent fashion at these concentrations.

**Figure 3:**
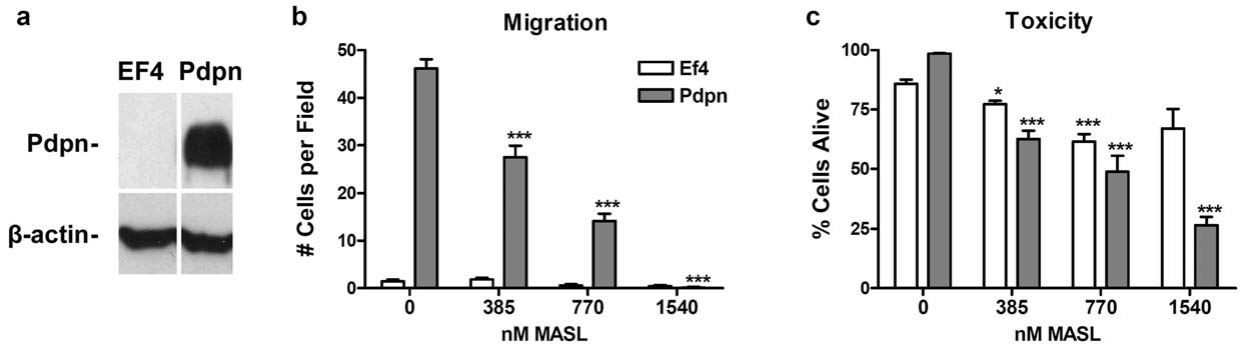
MASL targets PDPN to decrease cell migration and viability. (**a**) PDPN and β-actin were detected by Western blotting of protein (15 µg per lane) from nontransformed mouse embryonic cells (MEFs) transfected with cDNA encoding PDPN or empty parental vector (EF4) as indicated. (**b**) Wound healing migration assays were performed on confluent monolayers treated with concentrations of MASL as indicated. Data are shown as the number of cells that migrated into a 300×300 micron area along the center of the wound in 24 hours (mean + SEM, n = 7). (**c**) MASL toxicity was evaluated by Trypan blue staining of cells, and shown as the percent of live cells from each well (mean + SEM, n = 4). Single and triple asterisks indicate p<0.05 and p<0.001, respectively, compared to untreated controls.

To verify the functional relevance of MASL targeting PDPN on cell migration, we investigated its effects on nontransformed cells transfected with PDPN or empty parental vector. Since PDPN expression is sufficient to promote cell migration [5], [15], [30], nontransformed cells transfected with PDPN migrated several fold more than control transfectants (**Figure 3a and 3b**). In addition, MASL reduced the migration of these nontransformed PDPN transfected cells in a dose dependent fashion (**Figure 3b**). For example, 385 nM MASL decreased the migration of PDPN transfectants by over 40%.

As shown in **Figure 3c**, in addition to inhibiting cell migration, MASL was also toxic to PDPN expressing cells in a dose dependent fashion. In contrast, MASL did not inhibit the viability of empty vector transfectants in an equally dose dependent fashion. For example, 1540 nM MASL decreased Trypan blue exclusion of PDPN transfectants by over 70%, but control transfectants by only about 30%.

### MASL targets PDPN to inhibit melanoma cell growth and motility

**Figure 4:**
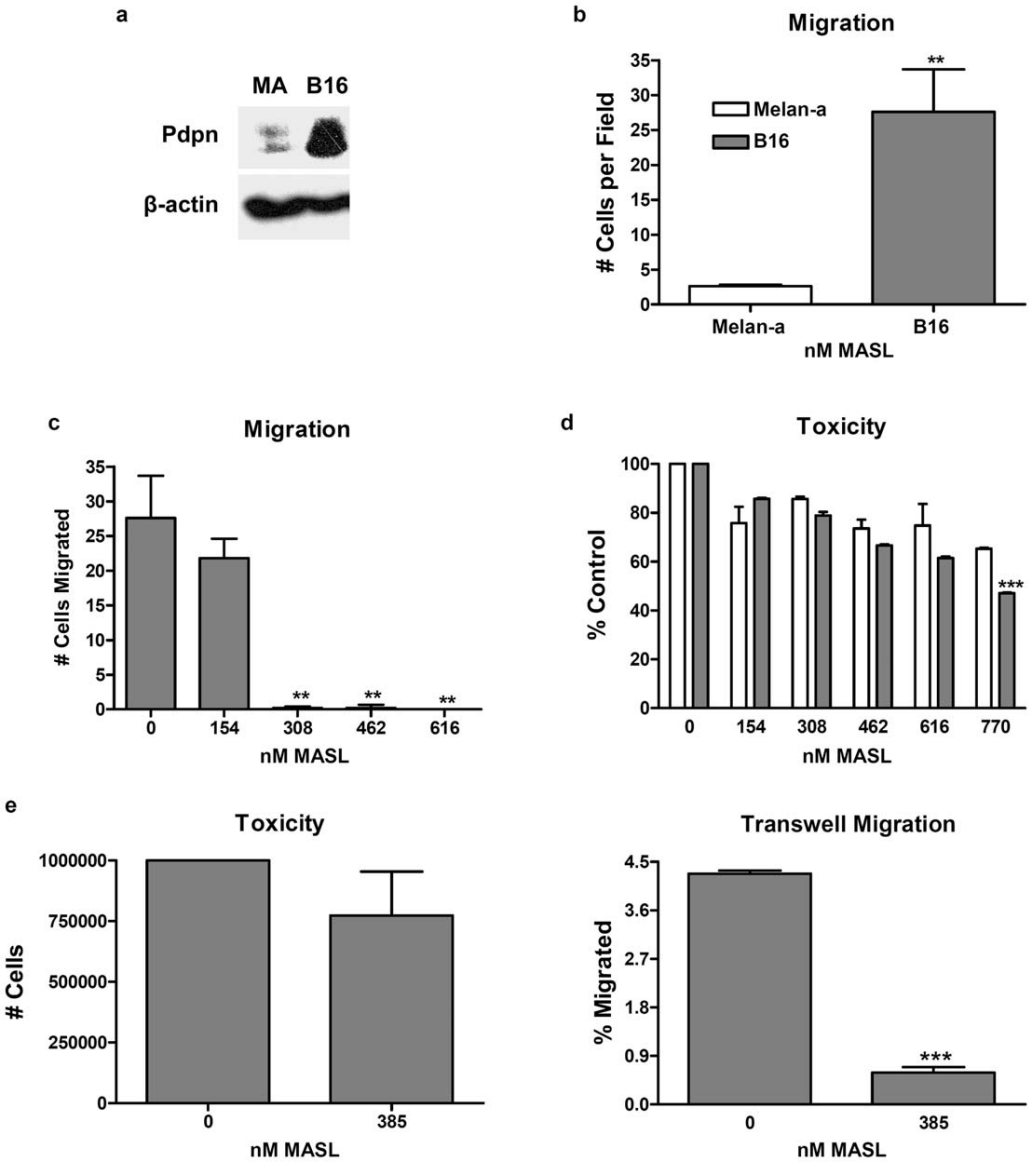
MASL inhibits melanoma cell motility and viability. (**a**) PDPN and β-actin were detected by Western blotting of protein (15 µg per lane) from Melan-a melanocytes and B16 melanoma cells. (**b**) Wound healing migration assays were performed on confluent MelanA and B16 monolayers. Data are shown as the number of cells that migrated into a 400×500 micron area along the center of the wound in 24 hours (mean + SEM, n = 5). (**c**) Wound healing migration assays were performed on confluent monolayers treated with MASL as indicated. Data are shown as the number of cells that migrated into a 400×500 micron area along the center of the wound in 24 hours (mean + SEM, n = 5). (**d**) Melan-a and B16 cells were treated with MASL, and cell viability was evaluated by Alamar blue assay. Data are shown as percent of nontreated cells (mean + SEM, n = 2). (**e**) Melanoma cell viability and Transwell migration assays were performed on 600,000 cells plated on cell culture inserts containing membranes with an 8 micron pore size in 6-well plates. Cell viability was evaluated by Alamar blue assay and shown as percent of nontreated cells, while Transwell migration was measured after 24****hours as the percent of cells found on the underside of the membrane (mean + SEM, n = 2). Double and triple asterisks indicate p<0.01 and p<0.001 compared to corresponding treatments of untreated cells or Melan-a cells in panels d and e, respectively.

Studies indicate that PDPN expression is strongly induced in about 80% of skin cancers [27], [28]. Consistent with its role in tumor cell invasion and metastasis, malignant B16 melanoma cells expressed higher levels of PDPN (**Figure 4a**) and migrated significantly better (**Figure 4b**) than syngeneic nontransformed Melan-a melanocytes. As shown in **Figure 4c**, MASL effectively suppressed melanoma cell migration at concentrations of 308 nM or less. In addition to inhibiting melanoma cell migration, MASL also inhibited melanoma cell growth in a dose responsive manner (**Figure 4d**). Moreover, MASL was significantly more toxic to B16 melanoma cells than Melan-a cells (p<0.001 by ANOVA).

Transwell chambers were used to further investigate the effects of MASL on melanoma cell growth and migration. As shown in **Figure 4e**, while 385 nM MASL was not significantly toxic to B16 melanoma cells (p>0.2 compared to controls), migration through 8 micron pores was decreased by over 40 fold. These data indicate that MASL suppressed melanoma cell migration prior to inhibiting cell viability.

**Figure 5:**
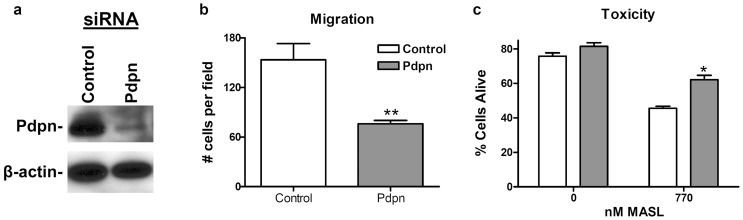
MASL targets PDPN to inhibit melanoma cell growth. (**a**) PDPN and β-actin were detected by Western blotting of protein (5 µg per lane) from B16 melanoma cells transfected with control siRNA or siRNA targeted against PDPN, as indicated. (**b**) Wound healing migration assays were performed on confluent B16 monolayers transfected with control siRNA or siRNA targeted against PDPN, as indicated. Data are shown as the number of cells that migrated into a 500×400 micron area along the center of the wound in 24 hours (mean + SEM, n = 4). (**c**) MASL toxicity was evaluated by Trypan blue staining of cells, and shown as the percent of live cells from each well (mean + SEM, n = 2). Single, double, and triple asterisks indicate p<0.05, p<0.01, and p<0.001, respectively, compared to nontransformed cells, untreated Src transformed cells, or control transfectants as indicated.

We employed siRNA to verify the effects of PDPN and MASL on melanoma cell growth and migration. PDPN siRNA effectively decreased B16 Pdpn expression levels (**Figure 5a**) and cell migration (**Figure 5b**). As shown in **Figure 5c**, this decreased PDPN expression resulted in a 25% decrease in MASL toxicity. These data indicate that while PDPN may not be the only receptor targeted by MASL on these melanoma cells, it is a functionally relevant receptor that can be targeted to prevent melanoma cell growth and migration.

### MASL may induce caspase independent necrosis of melanoma cells

Some lectins used as potential anticancer agents, including Mistletoe lectins, contain a distinct N-glycosidase motif that acts as a ribosome inactivating peptide (RIP) [45]. Since MASL does not contain this peptide, inhibition of tumor cell viability by MASL did not result from RIP activity. In general, cell death can occur by apoptosis, necrosis, or autophagy [53], [54]. MASL did not induce characteristics of autophagy such vacuolarization or the formation of autophagosomes, or characteristics of apoptosis including nuclear chromatin condensation, membrane blebbing, or chromosomal DNA fragmentation. Instead, MASL induced morphological changes typical of necrosis including cell swelling, membrane rupture, and nuclei fragmentation.

**Figure 6:**
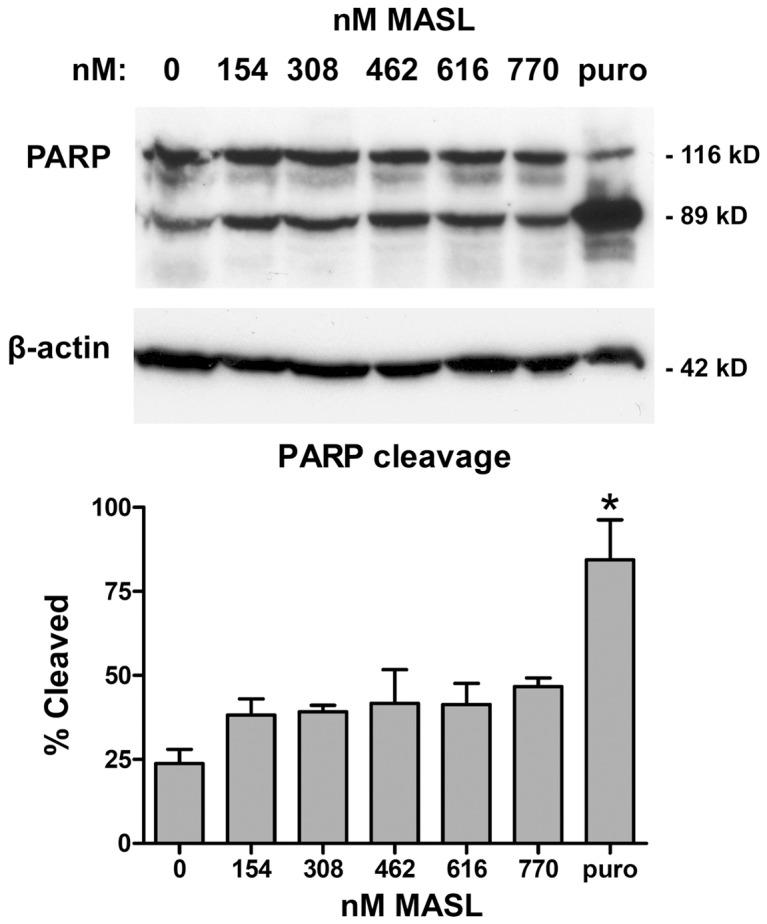
Effects of MASL on PARP cleavage. (**top**) PARP and β-actin were examined by Western blotting of protein from B16 melanoma cells treated for 24 hours with MASL or 37 µM puromycin as indicated. (**bottom**) Signal was quantitated by image densitometry (NIH Image) and shown as the percent of cleaved PARP compared to total PARP (mean + SEM, n = 2).

Apoptosis usually results from the induction of caspase activity which cleaves poly(ADP-ribose) polymerase (PARP) into an 89 kD fragment [55], [56]. As shown in **Figure 6**, while PARP cleavage was clearly detected in cells treated with puromycin which induces caspase mediated apoptosis [57], only slight PARP cleavage was seen in melanoma cells treated with MASL (p>0.2 by ANOVA). These observations suggest that MASL can reduce tumor cell growth by caspase independent necrotic mechanisms. This property may be particularly advantageous when applied to malignancies that resist apoptosis induced by classical cancer agents.

### Dietary MASL inhibits melanoma cell migration and tumorigenesis

**Figure 7:**
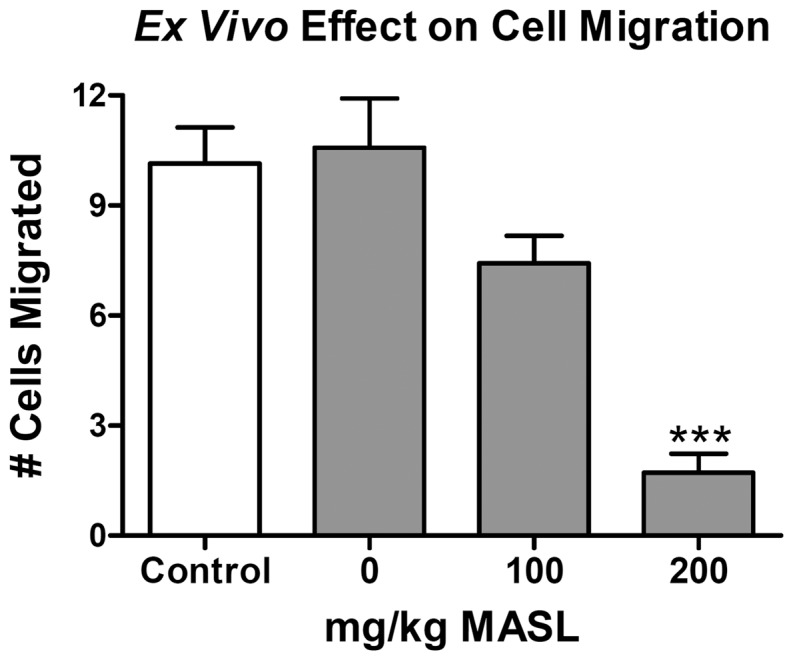
Dietary MASL bioactivity survives gastrointestinal proteolysis to enter the cardiovascular circulatory system and inhibit melanoma cell migration. Wound healing migration assays were performed on confluent monolayers of B16 melanoma cells treated with serum from mice fed MASL to achieve doses of 1, 100 mg/kg, or 200 mg/kg, or without mouse serum (controls) as indicated. Data are shown as the number of cells that migrated into a 200×300 micron area along the center of the wound in 24 hours (mean + SEM, n = 7). Triple asterisks indicate p<0.001 compared to controls.

As described above, some dietary lectins can survive digestive processing to combat cancer *in vivo* [43]–[46]. Serum was taken from mice fed MASL and then examined for its effects on melanoma cell migration *in vitro* to determine if this dietary lectin can survive gastrointestinal proteolysis to remain biologically active in the circulatory system. As shown in **Figure 7**, serum from mice fed 100 or 200 mg/kg MASL inhibited melanoma cell migration by about 30% or over 80% compared to serum from mice fed no MASL, respectively. These data indicate that dietary administration of MASL can result in biologically relevant levels of circulating product that are sufficient to inhibit melanoma cell migration.

**Figure 8:**
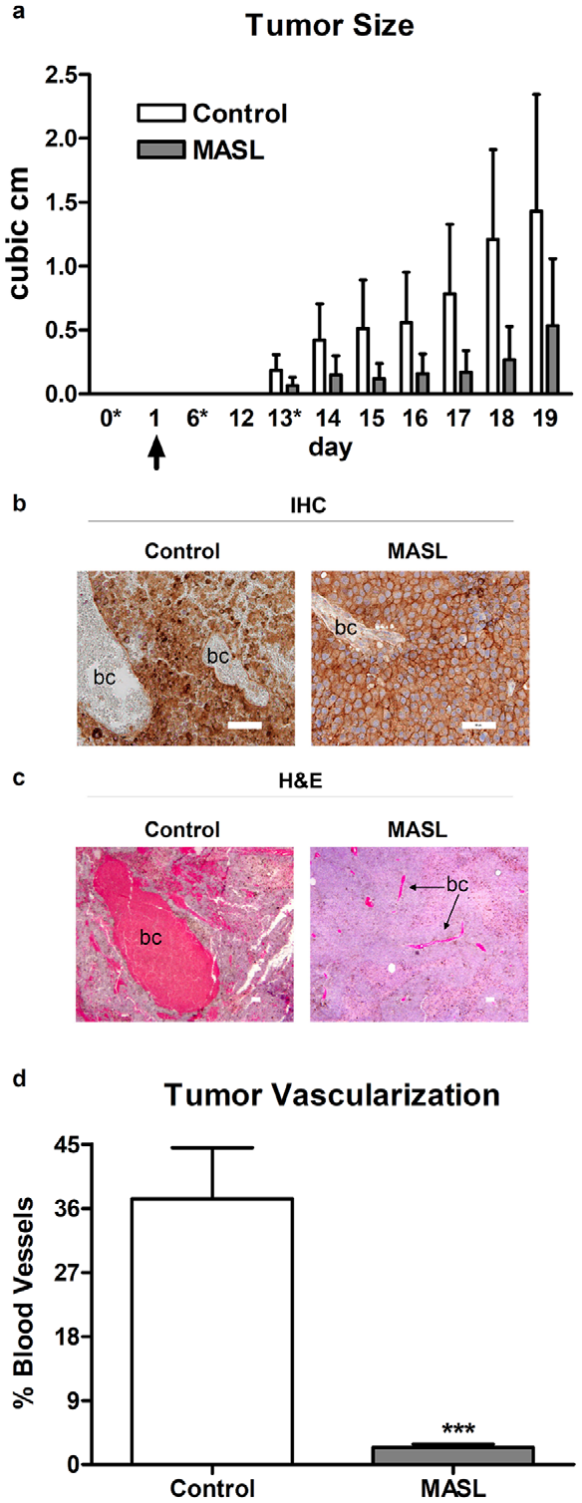
Dietary MASL inhibits melanoma growth *in vivo*. (**a**) Mice were fed MASL to achieve a dosage of 0 or 25 mg/kg once weekly (indicated by **asterisks**) and inoculated subcutaneously with B16 melanoma cells (100,000 cells per mouse on day indicated by **arrow**). Tumors were measured daily by caliper, and data is shown as cubic cm (mean + SEM, n = 4). (**b**) Tumors were evaluated for PDPN expression by IHC as indicated (bar  = 50 microns). “bc” indicates blood filled vascular spaces lined by tumor cells. (**c**) Tumors were examined by hematoxylin and eosin staining (H&E) to visualize vascularization and morphology. (**c**) Tumor vascularization was quantified as the percent of each field (0.8 mm2) occupied by blood vessels and shown as mean + SEM (n = 7).

Since MASL, or at least its biological activity, was resistant to gastrointestinal proteolysis we examined the effects of dietary MASL on tumor cell growth *in vivo*. As shown in **Figure 8a**, oral administration of 25 mg/kg of MASL once a week inhibited the subcutaneous growth of melanoma cells in mice by approximately 50% (p<0.05 by ANOVA). Moreover, we found no adverse effects on mouse health or physiology over the course of these experiments based on animal behavior, weight, and organ analysis after dissection.

As expected, PDPN expression was evident in melanoma cells of tumors *in vivo*. As shown in **Figure 8b**, PDPN expression was more intense in tumor cells from control animals than animals treated with MASL. In addition, tumor cells from MASL treated animals appeared more epithelial in nature, with a relatively flattened morphology and more restricted PDPN staining than tumors from untreated animals.

### Dietary MASL inhibits the formation of blood channels lined by melanoma cells

Although its molecular target has not been defined, Mistletoe lectin is used as an anticancer agent that can reduce tumor vascularization *in vivo* [46], [58], [59]. Aggressive melanoma cells are known for their ability to form blood vessels lined with tumor cells by a process referred to as “vasculogenic mimicry” [60]. Interestingly, as shown in **Figure 8b and 8c**, these blood filled vascular spaces lined by tumor cells were much smaller and less numerous in tumors from MASL treated mice than from control animals.

### Human melanoma cells express PDPN and respond to MASL

**Figure 9:**
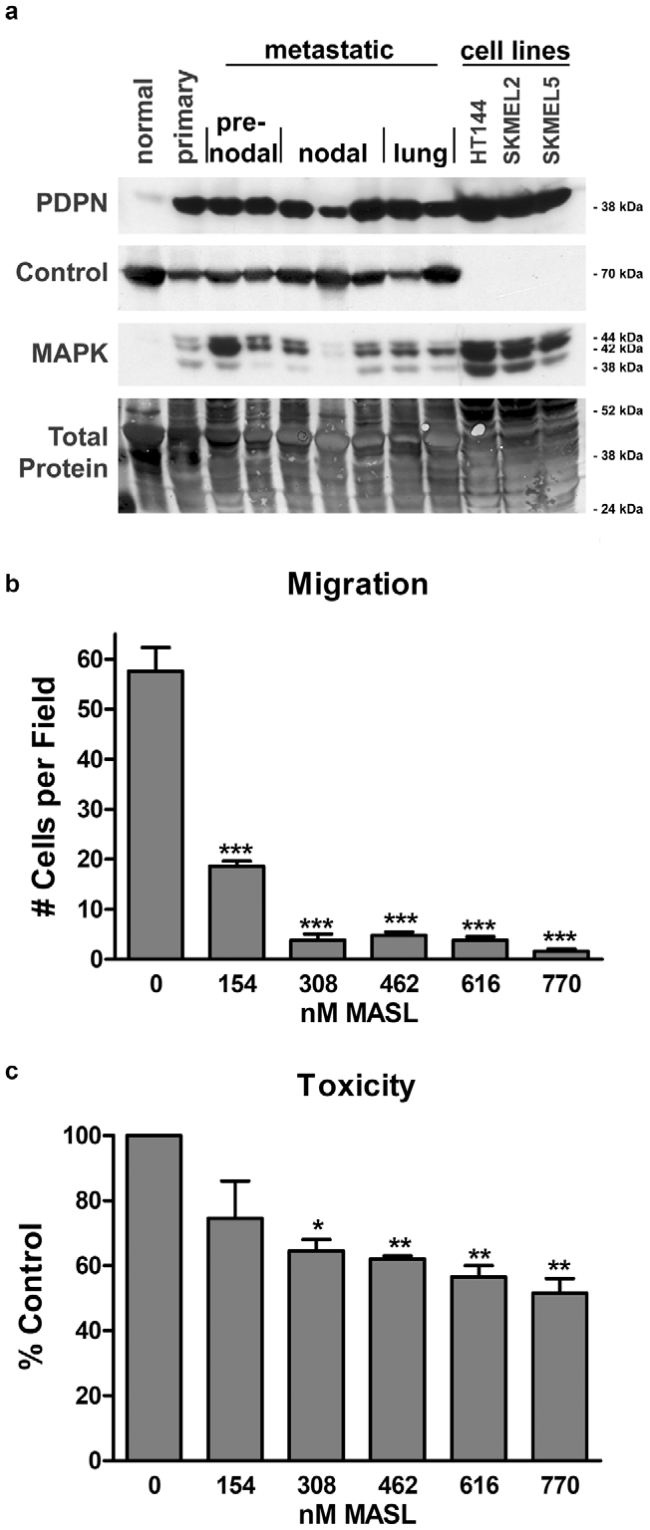
Human melanoma cells express PDPN and respond to MASL. (**a**) PDPN was detected by Western blotting of protein (15 µg per lane) from a variety of human specimens including normal skin, primary melanoma, melanoma in transit prior to lymph node metastasis, nodal metastasis, lung metastasis, and three melanoma cells lines (HT-144, SK-MEL-2, SK-MEL-5). Detection of MAPK and nonspecific bands by Western blotting and India ink staining of membranes is also shown to verify equal loading of these samples from different patients and cell lines. (**b**) Wound healing migration assays were performed on confluent HT-144 monolayers treated with MASL as indicated. Data are shown as the number of cells that migrated into a 400×500 micron area along the center of the wound in 24 hours (mean + SEM, n = 5). (**d**) HT-144 cells were treated with MASL, and cell viability was evaluated by Alamar blue assay. Data are shown as percent of nontreated cells (mean + SEM, n = 2). Single, double, and triple asterisks indicate p<0.05, p<0.01, and p<0.001 compared to untreated cells, respectively.

We examined cell lines and clinical specimens to further investigate PDPN expression and MASL sensitivity in human melanoma cells. As shown in **Figure 9a**, PDPN expression was drastically increased in primary and metastatic melanoma specimens from every patient that we examined. As with mouse melanoma cells, PDPN expression was also much higher in human melanoma cell lines than normal skin. In addition, as with mouse cells, MASL significantly suppressed human melanoma cell growth and migration at concentrations of less than 300 nM (**Figure 9b and 9c**). Moreover, as with mouse cells, MASL inhibited human melanoma cell motility prior to effecting cell growth. For example, 308 nM MASL inhibited HT-144 cell migration by over 10 fold, but cell growth by less than 40%.

### MASL consists of 2 subunits of identical primary amino acid sequence

**Figure 10:**
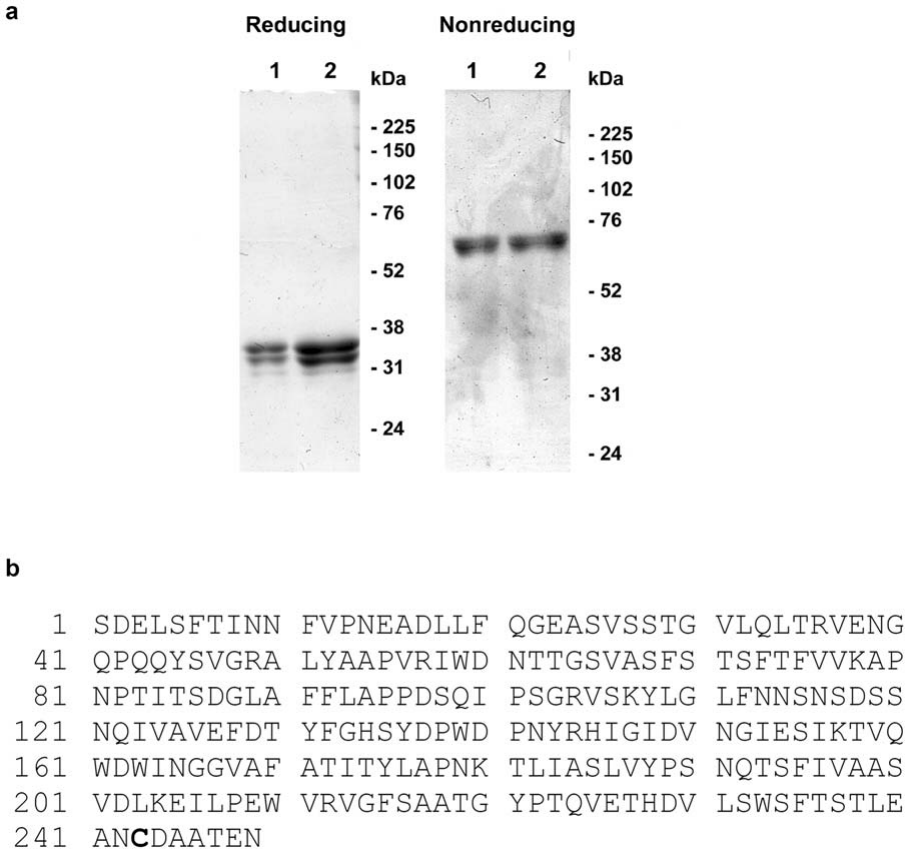
Analysis of MASL protein subunits. (**a**) MASL from Sigma (lane 1) or Sentrimed (lane 2) was analyzed by reducing and nonreducing SDS-PAGE. (**b**) Coomassie stained bands were excised, extracted and digested with trypsin, reduced and alkylated with iodoacetamide, and sequenced using data dependent acquisition by LC-MS/MS on a Thermo Scientific LTQ Orbitrap XL. All peptides were identical in primary sequence and contained a single cysteine at residue 243 (bold in figure).

Lectins are a complex family of proteins. MASL is a tetramer formed by 32 kD and 37 kD subunits. The 32 kD subunit is called *Maackia amurensis* hemagglutinin (MAH) and the 37 kD subunit is called *Maackia amurensis* leukoagglutinin (MAL) or *Maackia amurensis* mitogen (MAM) [52], [61], [62]. LC-MS-MS analysis shown in **Figure 10** indicates that both of these subunits consist of the same primary amino acid sequence that contains a single cysteine toward the carboxyl terminal at position 243. Unique molecular weights and other properties of these subunits are likely due to specific modifications including asparagine glycosylation and de-amidation events. The presence of a cysteine residue in MASL has been reported for the 37 kD subunit (MAL) [62]. In contrast, sequences reported for the 32 kD subunit (MAH) do not contain a cysteine, but contain a serine in its place [52], [61], [62]. Our finding that both 32 kD and 37 kD subunits contain this cysteine explains their dimerization into respective 64 kD and 74 kD subunits.

## Discussion

Lectins are promising anticancer agents that can be administered orally [47], [50]. For example, dietary Mistletoe lectin has been shown to inhibit lymphoma growth in mice and is used as an adjuvant therapy to treat various forms of cancer in people [43], [59]. Plant lectins are resistant to gastrointestinal proteolysis and breakdown by gut bacteria. Wang et al have shown that intact and bioactive peanut agglutinin (PNA) reaches concentrations of approximately 50nM in the serum of people within hours after ingesting 200g of peanuts [63]. Our data indicate that similar levels of lectin, or its bioactive digestion products, may be achieved from ingestion of MASL. These results also indicate that properties of MASL may be used to help develop reagents to prevent or combat melanoma.

Melanoma cell motility contributes to metastatic invasion that causes the vast majority of skin cancer deaths [4]. Robust PDPN expression was found 100% of the 8 melanoma samples examined from cancer patients in this study (see **Figure 9a**). These data are in contrast to previous studies reporting PDPN expression in less than 20% of spindle cell and other types of melanomas [64], [65]. This may be the result of different experimental methods. For example, the NZ1 antibody used here targets an approximately 12 amino acid epitope in the PDPN PLAG domain with a dissociation constant (*K_D_*) below 1 nM, while antibody used for these previous studies targets a larger epitope with lower affinity [66], [67]. However, PDPN staining seen in our patient samples may have been due to cancer associated fibroblasts (CAFs), which would not be distinguished from actual melanoma cells by Western blotting. Indeed, PDPN expression in CAFs has been correlated with tumor aggression in cancers including mammary carcinoma [68] and lung adenocarcinoma [69]–[71]. In any case, robust PDPN expression was seen in all of the melanoma cell lines that we examined, indicating that melanoma cells themselves express PDPN (see **Figures 4** **and** **9**).

Dietary lectins can block the action of endogenous pro-metastatic lectins (such as galectins or selectins) to inhibit tumor cell growth, and can be used as medicinal cancer treatments [37], [45]–[47]. While MASL has been shown to decrease viability of different types of transformed cells including melanoma [72], [73], its targets and effects on cell migration have not been reported. Our data indicate that MASL be used to target PDPN in order to combat tumor cell growth and migration. Consistent with PDPN acting as a functionally relevant target, these results indicate that MASL inhibits cell motility prior to inhibiting cell viability. For example, 308 nM MASL suppressed melanoma cell migration by over 99%, while inhibiting cell viability by about 20% within the same time period (see **Figure 4**).

Although some lectins may nonspecifically bind to many glycoproteins, experiments with cloned *Maackia amurensis* lectin indicate that it can accurately target specific glycoproteins expressed by human cells [74], [75]. Our data indicate that MASL, which has a high affinity for *O*-linked carbohydrate chains containing sialic acid [51], [52], targets PDPN to inhibit transformed cell growth and motility at nanomolar concentrations. Interestingly, *Maackia amurensis* has been used as a medicinal plant for several centuries to treat ailments including cancer in parts of Asia [76], [77]. However, as with many traditional medicines, clinical efficacy has been hindered by a lack of mechanistic understanding. Here, we describe how MASL can serve as a potent bioactive plant medicine that targets PDPN to combat cancer.

**Figure 11:**
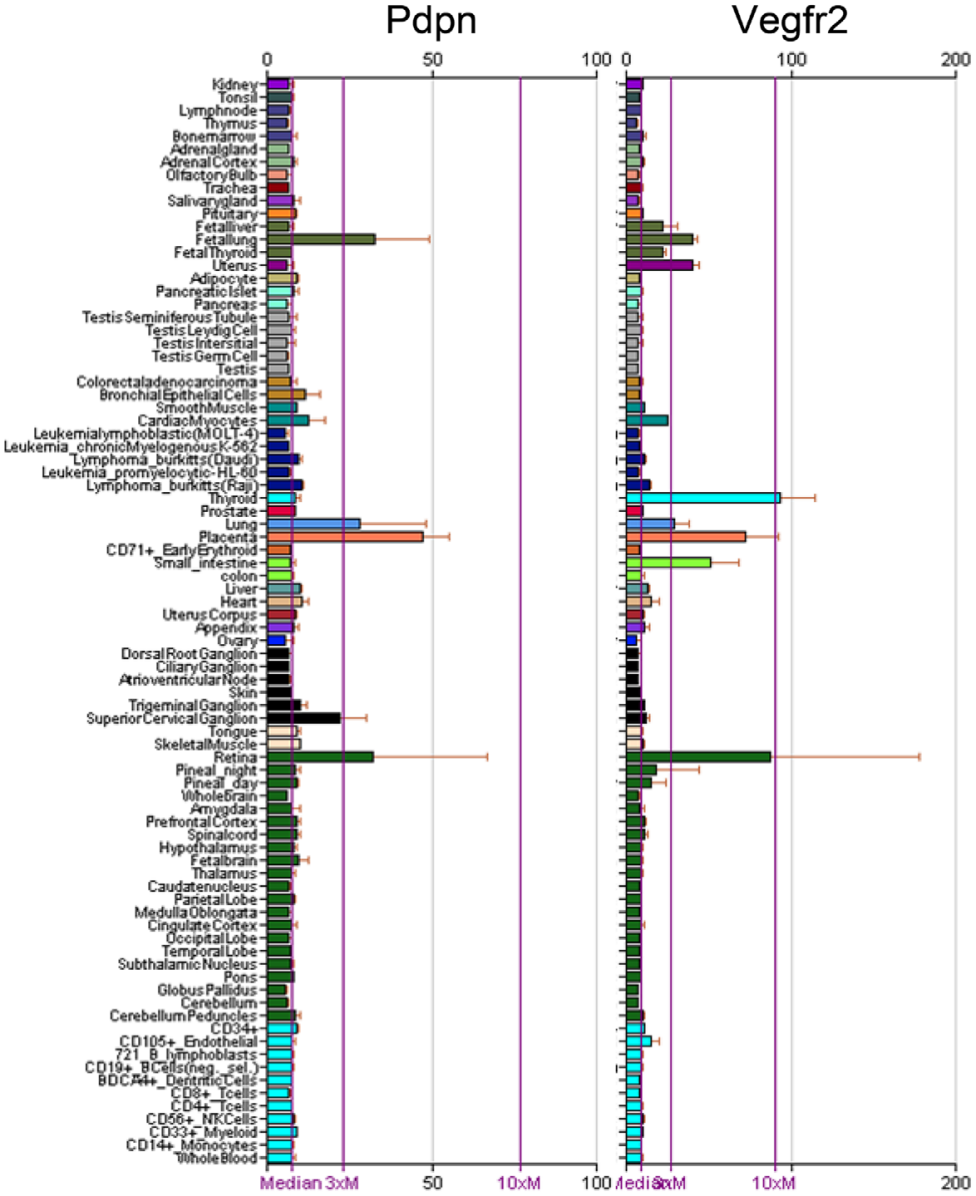
Comparison of Pdpn and Vegfr2 (Kdr) mRNA expression in human tissues. Values from Affymetrix probe sets representing Pdpn and Vegfr2 are shown as presented on BioGPS (http://biogps.gnf.org).

PDPN expression is relatively low in most normal cells, and found predominantly in tissues including retina, kidney podocytes, lymphatic endothelium, and lung alveolar epithelium [5], [78]. Indeed, as shown in **Figure 11**, PDPN expression in normal tissues is somewhat similar to that of VEGFR2 which serves as a useful chemotherapeutic target [79], [80]. Interestingly, PDPN and VEGFR2 are both receptors that promote cell migration and are suppressed during contact normalization by surrounding nontransformed cells in the microenvironment [15].

Our data demonstrate that MASL targets PDPN to inhibit tumor cell growth and motility. However, MASL is bound to target other receptors containing similar sialic acid motifs. In addition, like other reagents, MASL may produce “off target” effects in a clinical setting. However, lectin pharmacology is complex and MASL may undergo enzymatic modification to form bioactive compounds *in vivo*. For example, although legumes including lentils and peanuts contain significant amounts of agglutinating lectins, their ingestion does not result in toxic platelet aggregation [50]. Moreover, MASL caused no notable toxic effects on organ morphology or animal behavior in our studies which followed mice treated for about 3 weeks. As stated above, *Maackia amurensis* has been used as a medicinal plant for several centuries to treat ailments including cancer in parts of Asia [76], [77]. These treatments would result in oral administration of hundreds of mg of MASL per dosage, and have not resulted in reports of toxicity [81]. However, future studies will clearly be needed to further investigate the potential toxic effects of MASL or other lectins that may be considered for clinical use.

Taken together, results from this study suggest that lectins exemplified by MASL could significantly expand our limited arsenal of targeted cancer treatments, particularly anticancer agents that can be administered orally. This paradigm may lead to novel skin cancer treatments, and pave the way to treat other cancers with elevated PDPN expression levels including breast [20], [24], glioma [21], and oral cancer [25], [26]. However, as stated above, while PDPN served as the model target of this investigation, other receptors with α2,3-sialic acid residues are increased in melanoma and a variety of other cancers, and may also be targeted to some extent by MASL [10], [12]–[14], [42].

## Materials and Methods

### Ethics Statement

Use of mice to obtain serum was approved by Committee of Animal Experiment in Nagoya University Graduate School of Medicine (Approved ID: 23132). Mouse experimental protocols were approved by the University of Medicine and Dentistry School of Osteopathic Medicine Institutional Animal Care and Use Committee (Approved ID: 10350).

### Cell culture

Mouse embryonic cells transfected with v-Src, PDPN, or empty parental vectors have been previously described [15]. LA25 cells harboring a temperature sensitive v-Src construct were grown as described [82]. B16F10, SK-MEL-2, SK-MEL-5, and HT-144 melanoma cells were obtained from ATCC. Melan-a melanocytes were provided by Richard Niles [83]. Clones were not taken from any cell lines, thus minimizing potential effects of clonal variation. To knockdown Pdpn expression in B16 cells, cells were transfected with 200nM of nontargeting control siRNA (Dharmacon D-001810-10-05) or 200nM podoplanin siRNA (Dharmacon L-048117-01-0005) with Lipofectamine 2000 (Invitrogen 11668-027) as described [15]. For some experiments, cells were treated with MASL (Sigma L8025, Sentrimed MASL) or puromycin (Sigma P8833).

### Western blotting

Human tissue collected by the Honest Broker Program was excised and flash frozen in liquid nitrogen at the time of excision during surgery. Western blotting was performed as previously described [15], [84]–[86]. Protein was resolved by SDS-PAGE, transferred to Immobilon-P membranes (Millipore IH1079562), and incubated with antisera specific for PDPN (Sigma NZ1 P0085, Santa Cruz Biotechnology SC23564, or University of Iowa Developmental Studies Hybridoma Bank 8.1.1), active Src kinase (Cell Signaling Technology 2101), PARP (Cell Signaling Technology 9542), MAPK (Cell Signaling Technology 9102), or ß-actin (Sigma A1978). Primary antiserum was recognized by appropriate secondary antiserum conjugated to horseradish peroxidase and detected using Enhanced Chemiluminescence (Millipore WBKLS0500 or Thermoscientific 32106). After blotting, membranes were stained with India ink to verify equal loading and transfer.

### Immunofluorescence microscopy

Cells (300,000 per dish) were cultured on 35-mm poly-D-lysine–coated glass-bottomed culture dishes (MatTek P35GC-1.5-14-C) for 24 hours. MASL was conjugated to Hilyte Fluor TR (Anaspec 72052) and incubated with cells for 15 min at 37°C. Cells were fixed with 2% paraformaldehyde, permeabilized with 0.2% Triton X-100 in PBS for 10 minutes, washed thrice with 0.1% Tween 20 in PBS followed by 1% bovine serum albumin (BSA) in PBS for 30 minutes, incubated with PDPN antiserum (1∶100) overnight at 4°C, washed, and then labeled with goat anti-syrian hamster IgG conjugated to Alexa Fluor 488 (Molecular Probes A21110). Cells were visualized on a Zeiss Axiovert microscope as described [15], [85].

### Affinity precipitation

Cells were lysed in lysis buffer (20 mM Tris.Cl pH 7.4, 150 mM NaCl, 0.5% Triton X-100, 1mM PMSF) on ice for 30 min, clarified by centrifugation, diluted to 1 mg/ml in PBS supplemented with 1mM PMSF and 10 mM MgCl_2_, and incubated with MASL conjugated to agarose beads, or empty beads as controls (Thermo Scientific 20501), on ice for 3 hours. Beads were then washed 4 times with PBS, and eluted in SDS-PAGE sample buffer at 95°C for 5 minutes. Eluted protein was examined along with total cell lysates by Western blotting.

### Cell migration and toxicity assays

Cell monolayers were scratched and incubated with MASL for 24 hours to assess cell migration by wound healing assays, which were quantitated as the number of cells that entered an area of the wound indicated in Figure Legends as described [15], [84], [85]. Alamar blue (Invitrogen DAL1025) was added to cells 24 hours after MASL treatment, and incubated for an additional 8 hours to assay viability, which was detected by fluorescence measurements (ex/em 570/600 nm) as directed by the manufacturer (Invitrogen) in a Tecan GENios microplate fluorescence spectrophotometer. Cells were also stained with 0.2% trypan blue and counted with a hemocytometer to evaluate cytotoxicity. For Transwell migration assays, 600,000 cells were plated in 6-well cluster plates on cell culture inserts with an 8 micron pore size (Transwell-Clear, Costar) and grown for 24 hours. Cells were then released separately from the top of the membrane and the bottom of the membrane. Transwell migration was then quantitated as the percent of cells found on the underside of the membrane as described [15]. All experiments were performed on parallel cultures to control for variations in cell culture conditions.

### Ex vivo effects of dietary MASL on cell migration

Mice (C57BL/6) were fed with a 200 mg food pellet containing MASL to achieve doses of 0, 100, or 200 mg/kg 1 hour before blood was taken from subclavian vein. Blood was allowed to coagulate 1 hour at room temperature and clarified by centrifugation for 10 minutes at 20,000 g to obtain serum. This serum was added to cell culture medium (DMEM+10%FBS) to a final concentration of 20%. B16 cell monolayers were scratched, washed with DMEM, and then incubated with these media, or control medium not fortified with mouse serum, for 24 hours to assess their effects on cell migration by wound healing assays, which were quantitated as the number of cells that entered an area of the wound indicated in Figure Legends as described [15], [84], [85]. Use of mice to obtain serum was approved by Committee of Animal Experiment in Nagoya University Graduate School of Medicine (Approved ID: 23132).

### In vivo tumorigenesis

Mice (C57BL/6) were fed with a 200 mg food pellet containing MASL to achieve doses of 0 or 25 mg/kg once weekly, starting the day before subcutaneous inoculation with 100,000 B16 cells in 0.1 ml PBS. Tumor volumes were measured blindly by a trained physician with a single caliper daily. Mice were sacrificed 18 days after inoculation and dissected. Tumors were fixed in formalin, paraffin embedded, sectioned (5 microns), and processed for hematoxylin/eosin staining or immunohistochemistry to detect PDPN expression with 8.1.1 monoclonal antibody as described [15], [85], [87]. Samples were analyzed on a Zeiss Axiovert microscope with Axiovision software as described [15], [85]. Mouse experimental protocols were approved by the UMDNJ-SOM Institutional Animal Care and Use Committee (Approved ID: 10350).

### MASL mass spectroscopy

MASL subunits were resolved by reducing and nonreducing SDS-PAGE and stained by Coomassie dye. Bands were then extracted and digested with trypsin. Extracted protein was reduced and alkylated with iodoacetamide, and sequenced using data dependent acquisition by LC-MS/MS on a Thermo Scientific LTQ Orbitrap XL.

## References

[1] Jemal A, Bray F, Center MM, Ferlay J, Ward E, et al (2011) Global cancer statistics. CA Cancer J Clin. 61: 69–90. 21296855 10.3322/caac.20107

[2] Jemal A, Siegel R, Ward E, Hao Y, Xu J, et al (2008) Cancer statistics, 2008. CA Cancer J Clin 58: 71–96. 18287387 10.3322/CA.2007.0010

[3] Friedl P, Wolf K (2003) Tumour-cell invasion and migration: diversity and escape mechanisms. Nat Rev Cancer 3: 362–374. 12724734 10.1038/nrc1075

[4] Uong A, Zon LI (2010) Melanocytes in development and cancer. J Cell Physiol 222: 38–41. 19795394 10.1002/jcp.21935 PMC2783760

[5] Wicki A, Christofori G (2007) The potential role of podoplanin in tumour invasion. Br J Cancer 96: 1–5. 17179989 10.1038/sj.bjc.6603518 PMC2360213

[6] Wood ER, Truesdale AT, McDonald OB, Yuan D, Hassell A, et al (2004) A unique structure for epidermal growth factor receptor bound to GW572016 (Lapatinib): relationships among protein conformation, inhibitor off-rate, and receptor activity in tumor cells. Cancer Res 64: 6652–6659. 15374980 10.1158/0008-5472.CAN-04-1168

[7] Nelson MH, Dolder CR (2006) Lapatinib: a novel dual tyrosine kinase inhibitor with activity in solid tumors. Ann Pharmacother 40: 261–269. 16418322 10.1345/aph.1G387

[8] Bange J, Zwick E, Ullrich A (2001) Molecular targets for breast cancer therapy and prevention. Nat Med 7: 548–552. 11329054 10.1038/87872

[9] Los M, Roodhart JM, Voest EE (2007) Target practice: lessons from phase III trials with bevacizumab and vatalanib in the treatment of advanced colorectal cancer. Oncologist 12: 443–450. 17470687 10.1634/theoncologist.12-4-443

[10] Cui H, Lin Y, Yue L, Zhao X, Liu J (2011) Differential expression of the alpha2,3-sialic acid residues in breast cancer is associated with metastatic potential. Oncol Rep 25: 1365–1371. 21344161 10.3892/or.2011.1192

[11] Wang FL, Cui SX, Sun LP, Qu XJ, Xie YY, et al (2009) High expression of alpha 2, 3-linked sialic acid residues is associated with the metastatic potential of human gastric cancer. Cancer Detect Prev 32: 437–443. 19232843 10.1016/j.cdp.2009.01.001

[12] Inagaki Y, Tang W, Guo Q, Kokudo N, Sugawara Y, et al (2007) Sialoglycoconjugate expression in primary colorectal cancer and metastatic lymph node tissues. Hepatogastroenterology 54: 53–57. 17419230

[13] Shah MH, Telang SD, Shah PM, Patel PS (2008) Tissue and serum alpha 2-3- and alpha 2-6-linkage specific sialylation changes in oral carcinogenesis. Glycoconj J 25: 279–290. 18158621 10.1007/s10719-007-9086-4

[14] Chovanec M, Plzak J, Betka J, Brabec J, Kodet R, et al (2004) Comparative analysis of alpha2,3/2,6-linked N-acetylneuraminic acid and cytokeratin expression in head and neck squamous cell carcinoma. Oncol Rep 12: 297–301. 15254692

[15] Shen Y, Chen CS, Ichikawa H, Goldberg GS (2010) SRC induces podoplanin expression to promote cell migration. J Biol Chem 285: 9649–9656. 20123990 10.1074/jbc.M109.047696 PMC2843215

[16] Gandarillas A, Scholl FG, Benito N, Gamallo C, Quintanilla M (1997) Induction of PA2.26, a cell-surface antigen expressed by active fibroblasts, in mouse epidermal keratinocytes during carcinogenesis. Mol Carcinog 20: 10–18. 9328432 10.1002/(sici)1098-2744(199709)20:1<10::aid-mc3>3.0.co;2-m

[17] Nose K, Saito H, Kuroki T (1990) Isolation of a gene sequence induced later by tumor-promoting 12-O-tetradecanoylphorbol-13-acetate in mouse osteoblastic cells (MC3T3-E1) and expressed constitutively in ras-transformed cells. Cell Growth Differ 1: 511–518. 2088477

[18] Martin-Villar E, Yurrita MM, Fernandez-Munoz B, Quintanilla M, Renart J (2009) Regulation of podoplanin/PA2.26 antigen expression in tumour cells. Involvement of calpain-mediated proteolysis. Int J Biochem Cell Biol 41: 1421–1429. 19146981 10.1016/j.biocel.2008.12.010

[19] Navarro A, Perez RE, Rezaiekhaligh MH, Mabry SM, Ekekezie II (2010) Polarized Migration of Lymphatic Endothelial Cells is Critically Dependent on Podoplanin Regulation of Cdc42. Am J Physiol Lung Cell Mol Physiol. 10.1152/ajplung.00171.2010 21036919

[20] Wicki A, Lehembre F, Wick N, Hantusch B, Kerjaschki D, et al (2006) Tumor invasion in the absence of epithelial-mesenchymal transition: podoplanin-mediated remodeling of the actin cytoskeleton. Cancer Cell 9: 261–272. 16616332 10.1016/j.ccr.2006.03.010

[21] Cortez MA, Nicoloso MS, Shimizu M, Rossi S, Gopisetty G, et al (2010) miR-29b and miR-125a regulate podoplanin and suppress invasion in glioblastoma. Genes Chromosomes Cancer 49: 981–990. 20665731 10.1002/gcc.20808 PMC5559292

[22] Martin-Villar E, Fernandez-Munoz B, Parsons M, Yurrita MM, Megias D, et al (2010) Podoplanin Associates with CD44 to Promote Directional Cell Migration. Mol Biol Cell 21: 4387–4399. 20962267 10.1091/mbc.E10-06-0489 PMC3002391

[23] Martin-Villar E, Scholl FG, Gamallo C, Yurrita MM, Munoz-Guerra M, et al (2005) Characterization of human PA2.26 antigen (T1alpha-2, podoplanin), a small membrane mucin induced in oral squamous cell carcinomas. Int J Cancer 113: 899–910. 15515019 10.1002/ijc.20656

[24] Kono T, Shimoda M, Takahashi M, Matsumoto K, Yoshimoto T, et al (2007) Immunohistochemical detection of the lymphatic marker podoplanin in diverse types of human cancer cells using a novel antibody. Int J Oncol 31: 501–508. 17671675

[25] Kawaguchi H, El Naggar AK, Papadimitrakopoulou V, Ren H, Fan YH, et al (2008) Podoplanin: a novel marker for oral cancer risk in patients with oral premalignancy. J Clin Oncol 26: 354–360. 18202409 10.1200/JCO.2007.13.4072

[26] Yuan P, Temam S, El Naggar A, Zhou X, Liu DD, et al (2006) Overexpression of podoplanin in oral cancer and its association with poor clinical outcome. Cancer 107: 563–569. 16804930 10.1002/cncr.22061

[27] Liang H, Wu H, Giorgadze TA, Sariya D, Bellucci KS, et al (2007) Podoplanin is a highly sensitive and specific marker to distinguish primary skin adnexal carcinomas from adenocarcinomas metastatic to skin. Am J Surg Pathol 31: 304–310. 17255777 10.1097/01.pas.0000213388.47913.f1

[28] Schacht V, Dadras SS, Johnson LA, Jackson DG, Hong YK, et al (2005) Up-regulation of the lymphatic marker podoplanin, a mucin-type transmembrane glycoprotein, in human squamous cell carcinomas and germ cell tumors. Am J Pathol 166: 913–921. 15743802 10.1016/S0002-9440(10)62311-5 PMC1602360

[29] Kunita A, Kashima TG, Morishita Y, Fukayama M, Kato Y, et al (2007) The platelet aggregation-inducing factor aggrus/podoplanin promotes pulmonary metastasis. Am J Pathol 170: 1337–1347. 17392172 10.2353/ajpath.2007.060790 PMC1829466

[30] Scholl FG, Gamallo C, Vilaro S, Quintanilla M (1999) Identification of PA2.26 antigen as a novel cell-surface mucin-type glycoprotein that induces plasma membrane extensions and increased motility in keratinocytes. J Cell Sci 112 (Pt 24): 4601–4613. 10.1242/jcs.112.24.4601 10574709

[31] Cueni LN, Detmar M (2009) Galectin-8 interacts with podoplanin and modulates lymphatic endothelial cell functions. Exp Cell Res 315: 1715–1723. 19268462 10.1016/j.yexcr.2009.02.021 PMC3398156

[32] Christou CM, Pearce AC, Watson AA, Mistry AR, Pollitt AY, et al (2008) Renal cells activate the platelet receptor CLEC-2 through podoplanin. Biochem J 411: 133–140. 18215137 10.1042/BJ20071216 PMC2749330

[33] Suzuki-Inoue K, Kato Y, Inoue O, Kaneko MK, Mishima K, et al (2007) Involvement of the snake toxin receptor CLEC-2, in podoplanin-mediated platelet activation, by cancer cells. J Biol Chem 282: 25993–26001. 17616532 10.1074/jbc.M702327200

[34] Witz IP (2006) The involvement of selectins and their ligands in tumor-progression. Immunol Lett 104: 89–93. 16368149 10.1016/j.imlet.2005.11.008

[35] Witz IP (2006) Tumor-microenvironment interactions: the selectin-selectin ligand axis in tumor-endothelium cross talk. Cancer Treat Res 130: 125–140. 16610706

[36] Ingrassia L, Camby I, Lefranc F, Mathieu V, Nshimyumukiza P, et al (2006) Anti-galectin compounds as potential anti-cancer drugs. Curr Med Chem 13: 3513–3527. 17168720 10.2174/092986706779026219

[37] Hasan SS, Ashraf GM, Banu N (2007) Galectins – potential targets for cancer therapy. Cancer Lett 253: 25–33. 17207926 10.1016/j.canlet.2006.11.030

[38] Kato Y, Kaneko MK, Kunita A, Ito H, Kameyama A, et al (2008) Molecular analysis of the pathophysiological binding of the platelet aggregation-inducing factor podoplanin to the C-type lectin-like receptor CLEC-2. Cancer Sci 99: 54–61. 17944973 10.1111/j.1349-7006.2007.00634.x PMC11159596

[39] Nakazawa Y, Sato S, Naito M, Kato Y, Mishima K, et al (2008) Tetraspanin family member CD9 inhibits Aggrus/podoplanin-induced platelet aggregation and suppresses pulmonary metastasis. Blood 112: 1730–1739. 18541721 10.1182/blood-2007-11-124693

[40] Johnson KA, Brown PH (2010) Drug development for cancer chemoprevention: focus on molecular targets. Semin Oncol 37: 345–358. 20816505 10.1053/j.seminoncol.2010.05.012

[41] de Bono JS, Ashworth A (2010) Translating cancer research into targeted therapeutics. Nature 467: 543–549. 20882008 10.1038/nature09339

[42] Cerna A, Janega P, Martanovic P, Lisy M, Babal P (2002) Changes in sialic acid expression in the lung during intrauterine development of the human fetus. Acta Histochem 104: 339–342. 12553698 10.1078/0065-1281-00669

[43] Pryme IF, Dale TM, Tilrem P (2007) Oral mistletoe lectins: a case for their use in cancer therapy. Cancer Therapy 5: 287–300.

[44] Beuth J, Schneider B, Schierholz JM (2008) Impact of complementary treatment of breast cancer patients with standardized mistletoe extract during aftercare: a controlled multicenter comparative epidemiological cohort study. Anticancer Res 28: 523–527. 18383896

[45] Pusztai A, Bardocz S, Ewen SW (2008) Uses of plant lectins in bioscience and biomedicine. Front Biosci 13: 1130–1140. 17981618 10.2741/2750

[46] Pryme IF, Bardocz S, Pusztai A, Ewen SW (2006) Suppression of growth of tumour cell lines in vitro and tumours in vivo by mistletoe lectins. Histol Histopathol 21: 285–299. 16372250 10.14670/HH-21.285

[47] Liu B, Bian HJ, Bao JK (2010) Plant lectins: potential antineoplastic drugs from bench to clinic. Cancer Lett 287: 1–12. 19487073 10.1016/j.canlet.2009.05.013

[48] Augustin M, Bock PR, Hanisch J, Karasmann M, Schneider B (2005) Safety and efficacy of the long-term adjuvant treatment of primary intermediate- to high-risk malignant melanoma (UICC/AJCC stage II and III) with a standardized fermented European mistletoe (Viscum album L.) extract. Results from a multicenter, comparative, epidemiological cohort study in Germany and Switzerland. Arzneimittelforschung 55: 38–49. 15727163 10.1055/s-0031-1296823

[49] Icirsch A (2007) Successful treatment of metastatic melanoma with Viscum album extract (Iscador (R) M). J Alternative and Complementary Medicine 13: 443–445. 10.1089/acm.2007.6175 17532738

[50] De Mejia EG, Prisecaru VI (2005) Lectins as bioactive plant proteins: a potential in cancer treatment. Crit Rev Food Sci Nutr 45: 425–445. 16183566 10.1080/10408390591034445

[51] Imberty A, Gautier C, Lescar J, Perez S, Wyns L, et al (2000) An unusual carbohydrate binding site revealed by the structures of two Maackia amurensis lectins complexed with sialic acid-containing oligosaccharides. J Biol Chem 275: 17541–17548. 10747930 10.1074/jbc.M000560200

[52] Van Damme EJ, Van Leuven F, Peumans WJ (1997) Isolation, characterization and molecular cloning of the bark lectins from Maackia amurensis. Glycoconj J 14: 449–456. 9249142 10.1023/a:1018595300863

[53] Hotchkiss RS, Strasser A, McDunn JE, Swanson PE (2009) Cell death N Engl J Med. 361: 1570–1583. 10.1056/NEJMra0901217 PMC3760419 19828534

[54] Loos B, Engelbrecht AM (2009) Cell death: a dynamic response concept. Autophagy 5: 590–603. 19363298 10.4161/auto.5.5.8479

[55] Nicholson DW (1999) Caspase structure, proteolytic substrates, and function during apoptotic cell death. Cell Death Differ 6: 1028–1042. 10578171 10.1038/sj.cdd.4400598

[56] Edinger AL, Thompson CB (2004) Death by design: apoptosis, necrosis and autophagy. Curr Opin Cell Biol 16: 663–669. 15530778 10.1016/j.ceb.2004.09.011

[57] Theiss C, Mazur A, Meller K, Mannherz HG (2007) Changes in gap junction organization and decreased coupling during induced apoptosis in lens epithelial and NIH-3T3 cells. Exp Cell Res 313: 38–52. 17123514 10.1016/j.yexcr.2006.09.029

[58] Pryme IF, Bardocz S, Pusztai A, Ewen SW (1999) The growth of an established murine non-Hodgkin lymphoma tumour is limited by switching to a phytohaemagglutinin-containing diet. Cancer Lett 146: 87–91. 10656613 10.1016/s0304-3835(99)00242-6

[59] Pryme IF, Bardocz S, Pusztai A, Ewen SW (2002) Dietary mistletoe lectin supplementation and reduced growth of a murine non-Hodgkin lymphoma. Histol Histopathol 17: 261–271. 11820217 10.14670/HH-17.261

[60] Maniotis AJ, Folberg R, Hess A, Seftor EA, Gardner LM, et al (1999) Vascular channel formation by human melanoma cells in vivo and in vitro: vasculogenic mimicry. Am J Pathol 155: 739–752. 10487832 10.1016/S0002-9440(10)65173-5 PMC1866899

[61] Kawaguchi T, Matsumoto I, Osawa T (1974) Studies on hemagglutinins from Maackia amurensis seeds. J Biol Chem 249: 2786–2792. 4828319

[62] Yamamoto K, Konami Y, Irimura T (1997) Sialic acid-binding motif of Maackia amurensis lectins. J Biochem 121: 756–761. 9163528 10.1093/oxfordjournals.jbchem.a021650

[63] Wang Q, Yu LG, Campbell BJ, Milton JD, Rhodes JM (1998) Identification of intact peanut lectin in peripheral venous blood. Lancet 352: 1831–1832. 9851393 10.1016/S0140-6736(05)79894-9

[64] Buonaccorsi JN, Plaza JA (2012) Role of CD10, Wide-Spectrum Keratin, p63, and Podoplanin in the Distinction of Epithelioid and Spindle Cell Tumors of the Skin: An Immunohistochemical Study of 81 Cases. Am J Dermatopathol. 10.1097/DAD.0b013e318236b17f 22257901

[65] Yu H, Pinkus GS, Hornick JL (2007) Diffuse membranous immunoreactivity for podoplanin (D2-40) distinguishes primary and metastatic seminomas from other germ cell tumors and metastatic neoplasms. Am J Clin Pathol 128: 767–775. 17951198 10.1309/4GMREAULY257R3AY

[66] Ogasawara S, Kaneko MK, Price JE, Kato Y (2008) Characterization of anti-podoplanin monoclonal antibodies: critical epitopes for neutralizing the interaction between podoplanin and CLEC-2. Hybridoma (Larchmt.) 27: 259–267. 18707544 10.1089/hyb.2008.0017

[67] Kato Y, Vaidyanathan G, Kaneko MK, Mishima K, Srivastava N, et al (2010) Evaluation of anti-podoplanin rat monoclonal antibody NZ-1 for targeting malignant gliomas. Nucl Med Biol 37: 785–794. 20870153 10.1016/j.nucmedbio.2010.03.010 PMC2946889

[68] Pula B, Jethon A, Piotrowska A, Gomulkiewicz A, Owczarek T, et al (2011) Podoplanin expression by cancer-associated fibroblasts predicts poor outcome in invasive ductal breast carcinoma. Histopathology 59: 1249–1260. 22175904 10.1111/j.1365-2559.2011.04060.x

[69] Ito M, Ishii G, Nagai K, Maeda R, Nakano Y, et al (2012) Prognostic impact of cancer-associated stromal cells in stage I lung adenocarcinoma patients. Chest. 10.1378/chest.11-2458 22302300

[70] Hoshino A, Ishii G, Ito T, Aoyagi K, Ohtaki Y, et al (2011) Podoplanin-positive fibroblasts enhance lung adenocarcinoma tumor formation: podoplanin in fibroblast functions for tumor progression. Cancer Res 71: 4769–4779. 21610106 10.1158/0008-5472.CAN-10-3228

[71] Kawase A, Ishii G, Nagai K, Ito T, Nagano T, et al (2008) Podoplanin expression by cancer associated fibroblasts predicts poor prognosis of lung adenocarcinoma. Int J Cancer 123: 1053–1059. 18546264 10.1002/ijc.23611

[72] Wang H, Ng TB, Ooi VE, Liu WK (2000) Effects of lectins with different carbohydrate-binding specificities on hepatoma, choriocarcinoma, melanoma and osteosarcoma cell lines. Int J Biochem Cell Biol 32: 365–372. 10716633 10.1016/s1357-2725(99)00130-2

[73] Kapoor S, Marwaha R, Majumdar S, Ghosh S (2008) Apoptosis induction by Maackia amurensis agglutinin in childhood acute lymphoblastic leukemic cells. Leuk Res 32: 559–567. 17889364 10.1016/j.leukres.2007.08.007

[74] Maenuma K, Yim M, Komatsu K, Hoshino M, Tachiki-Fujioka A, et al (2009) A library of mutated Maackia amurensis hemagglutinin distinguishes putative glycoforms of immunoglobulin A1 from IgA nephropathy patients. J Proteome Res 8: 3617–3624. 19368344 10.1021/pr800816w

[75] Maenuma K, Yim M, Komatsu K, Hoshino M, Takahashi Y, et al (2008) Use of a library of mutated Maackia amurensis hemagglutinin for profiling the cell lineage and differentiation. Proteomics 8: 3274–3283. 18690646 10.1002/pmic.200800037

[76] Fedoreev SA, Kulish NI, Glebko LI, Pokushalova TV, Veselova MV, et al (2010) Maksar: A preparation based on amur maackia. Pharmaceutical Chemistry Journal 38: 605–610.

[77] Li X, Wang D, Xia MY, Wang ZH, Wang WN, et al (2009) Cytotoxic prenylated flavonoids from the stem bark of Maackia amurensis. Chem Pharm Bull (Tokyo) 57: 302–306. 19252325 10.1248/cpb.57.302

[78] Grimaldo S, Garcia M, Zhang H, Chen L (2010) Specific role of lymphatic marker podoplanin in retinal pigment epithelial cells. Lymphology 43: 128–134. 21226415 PMC4646604

[79] Ellis LM, Hicklin DJ (2008) VEGF-targeted therapy: mechanisms of anti-tumour activity. Nat Rev Cancer 8: 579–591. 18596824 10.1038/nrc2403

[80] Holmes K, Roberts OL, Thomas AM, Cross MJ (2007) Vascular endothelial growth factor receptor-2: structure, function, intracellular signalling and therapeutic inhibition. Cell Signal 19: 2003–2012. 17658244 10.1016/j.cellsig.2007.05.013

[81] “llustrated Manual of China Main Plants- the Family Leguminosae,”. Science Press, Beijing.

[82] Crow DS, Kurata WE, Lau AF (1992) Phosphorylation of connexin43 in cells containing mutant src oncogenes. Oncogene 7: 999–1003. 1315016

[83] Estler M, Boskovic G, Denvir J, Miles S, Primerano DA, et al (2008) Global analysis of gene expression changes during retinoic acid-induced growth arrest and differentiation of melanoma: comparison to differentially expressed genes in melanocytes vs melanoma. BMC Genomics 9: 478. 18847503 10.1186/1471-2164-9-478 PMC2572629

[84] Li X, Shen Y, Ichikawa H, Antes T, Goldberg GS (2009) Regulation of miRNA expression by Src and contact normalization: effects on nonanchored cell growth and migration. Oncogene 28: 4272–4283. 19767772 10.1038/onc.2009.278

[85] Shen Y, Jia Z, Nagele RG, Ichikawa H, Goldberg GS (2006) SRC uses Cas to suppress Fhl1 in order to promote nonanchored growth and migration of tumor cells. Cancer Res 66: 1543–1552. 16452211 10.1158/0008-5472.CAN-05-3152

[86] Alexander DB, Ichikawa H, Bechberger JF, Valiunas V, Ohki M, et al (2004) Normal cells control the growth of neighboring transformed cells independent of gap junctional communication and SRC activity. Cancer Res 64: 1347–1358. 14973064 10.1158/0008-5472.can-03-2558

[87] Li X, Jia Z, Shen Y, Ichikawa H, Jarvik J, et al (2008) Coordinate suppression of Sdpr and Fhl1 expression in tumors of the breast, kidney, and prostate. Cancer Sci 99: 1326–1333. 18422756 10.1111/j.1349-7006.2008.00816.x PMC11158056

